# Challenges in the Diagnosis of Iron Deficiency in Children Exposed to High Prevalence of Infections

**DOI:** 10.1371/journal.pone.0050584

**Published:** 2012-11-27

**Authors:** Ruth Aguilar, Cinta Moraleda, Llorenç Quintó, Montse Renom, Lázaro Mussacate, Eusebio Macete, Josep L. Aguilar, Pedro L. Alonso, Clara Menéndez

**Affiliations:** 1 Barcelona Centre for International Heath Research, Hospital Clínic, University of Barcelona, Barcelona, Spain; 2 CIBER Epidemiology and Public Health, Barcelona, Spain; 3 Manhiça Health Research Center, Maputo, Mozambique; 4 Department of Pathology, Hospital Clínic, University of Barcelona, Barcelona, Spain; University of Missouri-Kansas City, United States of America

## Abstract

**Background:**

While WHO guidelines recommend iron supplements to only iron-deficient children in high infection pressure areas, these are rarely implemented. One of the reasons for this is the commonly held view that iron supplementation increases the susceptibility to some infectious diseases including malaria. Secondly, currently used markers to diagnose iron deficiency are also modified by infections. With the objective of improving iron deficiency diagnosis and thus, its management, we evaluated the performance of iron markers in children exposed to high infection pressure.

**Methodology/Principal Findings:**

Iron markers were compared to bone marrow findings in 180 anaemic children attending a rural hospital in southern Mozambique. Eighty percent (144/180) of the children had iron deficiency by bone marrow examination, 88% (155/176) had an inflammatory process, 66% (119/180) had moderate anaemia, 25% (45/180) severe anaemia and 9% (16/180) very severe anaemia. Mean cell haemoglobin concentration had a sensitivity of 51% and specificity of 71% for detecting iron deficiency. Soluble transferrin receptor (sTfR) and soluble transferrin receptor/log ferritin (TfR-F) index (adjusted by C reactive protein) showed the highest areas under the ROC curve (AUC^ROC^) (0.75 and 0.76, respectively), and were the most sensitive markers in detecting iron deficiency (83% and 75%, respectively), but with moderate specificities (50% and 56%, respectively).

**Conclusions/Significance:**

Iron deficiency by bone marrow examination was extremely frequent in these children exposed to high prevalence of infections. However, even the best markers of bone marrow iron deficiency did not identify around a quarter of iron-deficient children. Tough not directly extrapolated to the community, these findings urge for more reliable, affordable and easy to measure iron indicators to reduce the burden of iron deficiency anaemia in resource-poor settings where it is most prevalent.

## Introduction

Iron deficiency (ID) is the most common and widespread nutrient deficiency, affecting approximately two billion people worldwide and resulting in over 500 million cases of anaemia [1], [2]. In sub-Saharan Africa, the prevalence of iron-deficiency anaemia (IDA) is estimated around 60% [1], [2], with 40 to 50% of children under five years of age in developing countries being iron deficient [3]. ID has been estimated to cause around 800,000 deaths and 35,057,000 disability adjusted life years lost annually [2], with the greatest toll in South-East Asia and Africa [1], [4].

By six months of age there is a physiological depletion of the iron stores that were accumulated by the foetus in the last months of pregnancy. If the infant’s diet does not provide enough iron, there is a significant risk to develop IDA. This physiological iron deficiency is often exacerbated by the early introduction of weaning foods [4], that frequently contain iron absorption inhibitors [5]. Iron deficiency may also be worsened by intestinal chronic blood loss from intestinal parasitic infections [3], [6]. All these determinants are frequent in developing countries, leading to a prevalence of ID that may reach more than 30% by 12 months of age [7]. Because IDA tends to develop slowly, adaptation occurs and the disease can go unrecognized for long periods, yet having an important impact on the children’s physical and cognitive development [8].

The controversy around the risk-benefit ratio of giving iron supplements to individuals exposed to malaria is still unresolved [9], [10]. While a recent Cochrane review on this issue concluded that “iron supplementation does not adversely affect children living in malaria-endemic areas and should not be withheld from them” [11], the current WHO guidelines on iron supplementation to children exposed to malaria and high prevalence of infections recommend “against universal iron supplementation for children under the age of two years living in malaria-endemic areas” [12], [13]. Moreover, screening to identify iron-deficient children is recommended “with directed treatment of iron-deficient children only” [13]. This inconsistency between the evidence and what it is actually recommended is leading to different interpretations by policy makers and health personnel, and a lack of implementation of policies to prevent a significant global health problem.

**Table 1:** Demographic and clinical characteristics of the study participants.

Characteristics				Result	
Age (months)*				22.06 (13.67)	
Gender		Male		102 (57%)	
		Female		78 (43%)	
Fever				163 (91%)	
Wasted (WAZ<−2)				88 (49%)	
Stunted (HAZ<−2) (n = 179)				56 (31%)	
Haemoglobin*				7.73 (1.97)	
Degree of anaemia		Moderate		119 (66%)	
		Severe		45 (25%)	
		Very severe		16 (9%)	
Inflammation (n = 176)				155 (88%)	
*P. falciparum* (n = 170)			74 (44%)		
Clinical Malaria (n = 170)			73 (43%)		
HIV (n = 164)			40 (24%)		
Parvovirus B19			15 (8%)		
Epstein-Barr virus			56 (31%)		
Bacteraemia (n = 173)			13 (8%)		
α-Thalassaemia (n = 41)			32 (78%)		
Bone marrow iron content	Absent		54 (30%)		
		Diminished		90 (50%)	
		Normal		14 (8%)	
		Increased		22 (12%)	

* Arithmetic Mean (SD).

N = 180 and results expressed as n (%) unless otherwise indicated.

Abbreviations: HAZ, height for age Z score; Hb, haemoglobin; HIV, human immunodeficiency virus; WAZ, weight for age Z score.

The diagnosis of IDA may be suggested by some signs and symptoms, but specially by blood tests indicating low haemoglobin, ferritin, and plasma iron levels. However, it has long been recognized that in developing countries interpretation of these and other biochemical tests is limited by the confounding effects of infection, inflammation and malnutrition [14], [15], [16], [17]. Thus, precisely where IDA is most common, it is also more difficult to diagnose and therefore treat.

**Table 2:** Proportion of children classified as iron deficient using internationally accepted cut-off values of iron markers.

			Iron deficient	
Iron marker	Obs.	Normal levels	n	%
**Ferritin (ng/ml)**	173	30–300	21	12
**Ferritin (ng/ml) by CRP**	173		15	9
CRP<1 mg/dl	21	12–300		
CRP≥1 mg/dl	152	30–300		
**Ferritin (ng/ml) by age**	173		1	1
3–5 months	6	50–200		
>5 months	167	7–140		
**sTfR (mg/l)**	163	0.83–1.76	124	76
**TfR-F index**	163	≤1.5	57	35
**TfR-F index by CRP**	163		63	39
CRP<1 mg/dl	17	≤1.5		
CRP≥1 mg/dl	146	≤0.8		
**Plasma iron (µg/dl)**	176	22–150	114	65
**Transferrin (g/l)**	176	2.0–3.85	1	1
**Transferrin saturation (%)**	176	16–45	135	77
**TIBC (mg/l)**	176	1–4	24	14
MCHC (g/dl)*	173	32.0–36.8	81	47
**MCV (fl) by age**	174		87	50
<2 years	110	70–91		
≥2 years	64	73–89		

Abbreviations: CRP, C reactive protein; MCHC, mean cell haemoglobin concentration; MCV, mean cell volume; Obs, observations; sTfR, soluble transferrin receptor; TfR-F index, transferrin-ferritin index; TIBC, total iron binding capacity.

A reliable, non-invasive tool for the assessment of ID in these populations remains elusive. The use of the ratio of soluble transferrin receptor to log ferritin concentrations (sTfR/log ferritin index) has been advocated to assess iron status [18]. However, this index is also limited because its parameters are influenced by the erythropoietic activity and inflammation [19], [20]. Moreover, we found that malaria infection was associated with a significant increase in sTfR plasma levels, even higher than those observed in IDA, thus questioning the role of sTfR levels in the diagnosis of IDA in individuals exposed to malaria [16].

**Table 3:** Sensitivity, specificity and accuracy of internationally accepted cut-off values of iron markers to identify iron stores deficiency using bone marrow iron content as “gold standard”.

Iron marker	True		False				
	Pos	Neg	Pos	Neg	Sensitivity(%)	Specificity(%)	Accuracy(%)
Ferritin (ng/ml)	21	35	0	117	15	100	32
Ferritin (ng/ml) 1	15	35	0	123	11	100	29
Ferritin (ng/ml) 2	1	35	0	137	1	100	21
sTfR	107	17	17	22	83	50	76
TfR-F index	54	31	3	75	42	91	52
TfR-F index 3	97	19	15	32	75	56	71
Plasma iron	98	19	16	43	70	54	66
Transferrin	1	35	0	140	1	100	20
Transferrin saturation	114	14	21	27	81	40	73
TIBC	24	35	0	117	17	100	34
MCHC	71	24	10	68	51	71	55
MCV 4	69	16	18	71	49	47	49

1 By C reactive protein (CRP): <12 ng/ml if CRP<1 mg/dl, and <30 ng/ml if CRP≥1 mg/dl.

2 By age: <50 ng/ml in children 3–5 months of age, and <7 ng/ml in children >5 months of age.

3 By CRP: >1.5 if CRP<1 mg/dl, and >0.8 if CRP≥1 mg/dl.

4 By age: <70 fl in children<2 years of age, and <73 fl in children ≥2 years of age.

Abbreviations: MCHC, mean cell haemoglobin concentration; MCV, mean cell volume; Neg, negative; Pos, positive; sTfR, soluble transferrin receptor; TfR-F index, transferrin-ferritin index; TIBC, total iron binding capacity.

Until now, the microscopic examination of Perl’s Prussian blue stained bone marrow aspirate remains the “gold standard” for the assessment of iron stores [21]. However, this is an invasive procedure and not logistically feasible in most settings where the diagnosis of ID is both most needed and problematic.

**Table 4:** AUC^ROC^ values for iron markers to identify children with iron stores deficiency*.

Iron marker	Area under ROC curve	(95% CI)	p-value
Ferritin	0.70	(0.61, 0.79)	0.0268
sTfR	0.75	(0.66, 0.84)	0.0059
TfR-F index	0.76	(0.68, 0.85)	0.0024
Plasma iron	0.64	(0.53, 0.75)	0.1584
Transferrin	0.71	(0.61, 0.81)	0.0298
Transferrin saturation	0.70	(0.60, 0.80)	0.0326
TIBC	0.71	(0.61, 0.81)	0.028
MCHC	0.59	(0.49, 0.70)	0.3382
MCV	0.55	(0.43, 0.66)	0.6311

* This analysis includes only children with results for all iron markers (n = 159).

Abbreviations: CI, confidence interval; MCHC, mean cell haemoglobin concentration; MCV, mean cell volume; Obs, observations; ROC, receiver operating characteristic; sTfR, soluble transferrin receptor; TfR-F index, transferrin-ferritin index; TIBC, total iron binding capacity.

**Figure 1:**
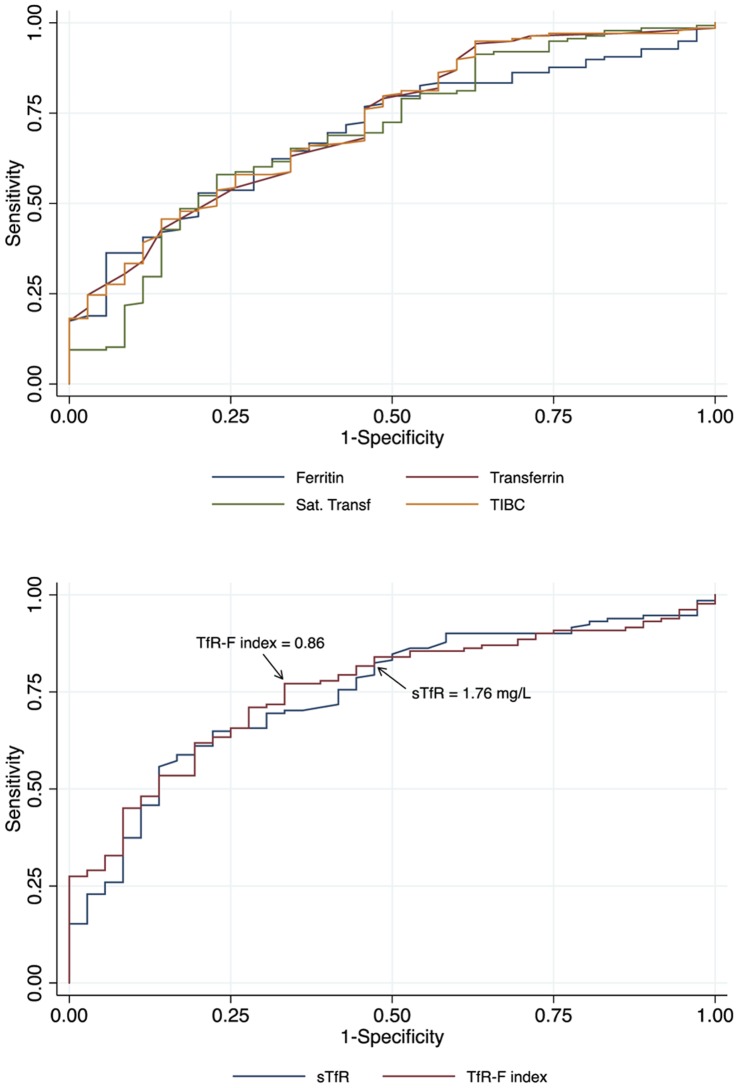
Receiver operating characteristic curves of the iron markers in the identification of iron stores deficiency. Cut-off values for sTfR and TfR-F index with the highest sensitivity to detect iron deficiency maintaining the specificity≥50% are indicated with arrows. Abbreviations: Sat. Transf., transferrin saturation; sTfR, soluble transferrin receptor; TfR-F index, transferrin-ferritin index; TIBC, total iron binding capacity.

A previous study among severely anaemic Malawian children comparing various iron markers against bone marrow iron content found that TfR-F index was the best predictor of bone marrow iron stores deficiency (sensitivity 74% and specificity 73%) [22]. However, even using this index as a proxy for ID, a significant number of iron deficient children would not be diagnosed and thus receive adequate treatment. On the other hand, evaluation of the performances of iron markers in other well defined populations from highly infectious settings is needed to know if they could be extrapolated. In order to contribute to improving the diagnosis of ID in children exposed to high infection pressure, we have evaluated the sensitivity and specificity of currently used iron markers using bone marrow iron content as the “gold standard” in Mozambican children with several degrees of anaemia.

## Materials and Methods

### Ethics Statement

The study protocol was approved by the National Mozambican Ethics Committee and the Hospital Clínic of Barcelona Ethics Review Committee. Parents-guardians were informed of the goals, procedures, benefits and risks of taking a bone marrow sample from their child, and it was never offered to them any financial or material inducement to agree on it. They were also given the choice of consenting to the participation of their child in the anaemia case-control study but refusing to bone marrow sample donation for the iron biomarkers study here presented. All the explanations were given in Portuguese (the National language) and when required in Changana (the local language). The parents-guardians of all children included in the study provided written informed consent.

### Study Site

The study was carried out at the Centro de Investigação em Saúde de Manhiça (CISM) in Manhiça District, southern Mozambique. The characteristics of the area have been described in detail elsewhere [23], [24], [25]. Malaria transmission of moderate intensity is perennial with some seasonality. More than 95% of the malaria infections are due to *Plasmodium falciparum* [26]. Adjacent to the CISM is the Manhiça District Hospital (MDH), a 110 bed health facility. The main causes of hospital attendance and admission among children in the area are pneumonia [27], malaria [25], anaemia [24], malnutrition and HIV-related diseases (unpublished data). HIV prevalence in pregnant women was 29% in 2010 [28].

### Study Participants and Procedures

The study was undertaken as part of a case-control study on the aetiology and risk factors of anaemia in children less than 5 years of age. Children aged 1 to 59 months, attending the MDH emergency department between October 2008 to August 2010 with anaemia (haemoglobin (Hb) <11 g/dl), and with no history of blood transfusion in the preceding 4 weeks, were recruited as cases if their parents-guardians gave written informed consent. Haemoglobin concentration was measured at the time of recruitment by the HemoCue® system (HemoCue® HB 201^+^, Änghelom, Sweden). A complete clinical examination was performed and the information was entered onto standardized questionnaires together with demographic data. Four ml of venous blood were collected by venipuncture for malaria parasitaemia examination, bacterial culture, full blood count and biochemical and molecular determinations. Participating children were offered voluntary HIV counselling and testing. A bone marrow aspiration was performed from the anterior-superior iliac crest or the tibia, under conscious sedation with parenteral ketamine, atropine and diazepam [29], [30], [31]. Bone marrow aspirates were not performed in children <3 months of age or with medical counter-indications such as severe respiratory distress, history of seizures, suspected intracranial hypertension, or any risk at the discretion of the responsible clinician. There were no adverse effects associated to bone marrow biopsy, however there were three adverse effects associated to sedation. One child presented ***bronchial hyper-secretion and bone marrow aspirate was then not performed. Two other children vomited during the aspirate, also due to the administration of sedatives.*** Resuscitation equipment was always available during the procedure. All children received treatment according to their clinical condition and following national guidelines.

### Laboratory Methods

A complete blood count was performed on an automated haematology analyzer Sysmex XT-2000i (Sysmex Corporation, Randburg, South Africa). *P. falciparum* parasites were identified by microscopy of thick and thin Giemsa-stained blood films [32]. *P. falciparum*-specific real time quantitative PCR (qPCR) was performed on microscopically negative samples [33]. HIV status was assessed using the Determine HIV-1/2 Rapid Test (Abbott Laboratories, Abbott Park, IL) and positive results were confirmed by the Uni-Gold Rapid Test (Trinity Biotech Co., Wicklow, Ireland). For children <18 months who were positive by both HIV rapid tests and for cases with discordant results, HIV infection was confirmed using the HIV-1 DNA-PCR kit (Roche Molecular Systems, Branchburg, NJ, USA) [34], [35]. Blood was cultured using an automated system (BACTEC® 9050; Becton-Dickinson, Franklin Lake, NJ, USA) [36], [37]. Epstein-Barr virus (EBV) and Parvovirus B19 (PV-B19) were identified by real time qPCR using the Artus EBV RG PCR and the Artus Parvo B19 RG PCR kits (QIAGEN), respectively. Diagnosis of α-thalassaemia (3.7 kb deletion) was performed by the GAP-PCR [38] in 121 anaemic children of the case-control study, of which only 41 had analysable bone marrow material to be included in this analysis.

Plasma was stored at −80°C until iron biochemical markers were determined. Plasma iron, transferrin and C reactive protein (CRP) were measured in an ADVIA 2400 analyser (Siemens Healthcare, Barcelona, Spain). Ferritin was measured in an ADVIA Centaur analyser (Siemens Healthcare, Barcelona, Spain). sTfR was measured in a BN-II nephelometer (Dade-Siemens Healthcare, Barcelona, Spain). Transferrin saturation and TIBC were calculated from the transferrin and iron data according to a standard formula [39].

Bone marrow smears were air-dried, fixed with formaldehyde vapour and stained by the Perls' Prussian blue method using clorhidric solution of potassium ferrocyanide and Harris haematoxylin. Bone marrow iron content was semi-quantitatively estimated classifying the amount of blue stained haemosiderin perls in bone marrow fragments (aggregates of bone marrow cells) according to 4 categories: 0 (absent), 1 (diminished), 2 (normal) and 3 (abundant) [40]. The categories 0 and 1 were considered indicative of iron stores deficiency [40]. The quantification of haemosiderin perls was performed by an experienced haematologist blinded to clinical and laboratory data (JLA).

### Definitions and Cut-off Values

Moderate anaemia was defined as an Hb concentration <11 and ≥7 g/dl, severe anaemia as Hb <7 and ≥5 g/dl, and very severe anaemia as Hb <5 g/dl. *P. falciparum* infection was defined as presence of asexual parasites in blood detected either by microscopy or real time qPCR. Clinical malaria was defined as the above plus fever (axillary temperature ≥37°C) or history of fever in the preceding 24 hours. Inflammation was defined as CRP≥1 mg/dl [41]. Wasting was defined as weight for height/length Z-score<−2 standard deviations (SD) and stunting as height for age Z-score<−2 SD.

Internationally accepted cut-off values for the iron status markers used in the analysis were as follows: i) ferritin <12 ng/ml if CRP<1 mg/dl, and <30 ng/ml if CRP≥1 mg/dl [42]; <50 ng/ml in children 3–5 months of age, and <7 ng/ml in children >5 months of age [43]; ii) plasma iron <22 µg/dl [43]; iii) plasma transferrin >3.85 g/l [43]; iv) TIBC≥4 mg/l [43]; v) transferrin saturation <16% [42]; vi) sTfR≥1.76 mg/l (laboratory reference); vii) TfR-F index [sTfR/log ferritin] >1.5 if CRP<1 mg/dl, and >0.8 if CRP≥1 mg/dl [18], [44]; viii) MCHC<32 g/dl [42]; ix) MCV<70 fl in children <2 years of age, and <73 fl in children ≥2 years of age [45].

### Statistical Analysis

The prevalence of iron stores deficiency diagnosed by each marker was estimated as the percentage of children with a value of that marker outside the internationally accepted normal range. The classification of ID by each marker was compared with the classification obtained using the “gold standard” (iron content in the bone marrow) to determine sensitivity, specificity and accuracy of each of them. To visualize the efficacy of each marker to detect ID, Receiver Operating Characteristics (ROC) curves were constructed and the areas under the resulting ROC curves (AUC^ROC^) were calculated [46]. When a marker does not identify ID the ROC curve lies close to the diagonal and the AUC^ROC^ is close to 0.5. Therefore an AUC^ROC^ not statistically different to 0.5 indicates an ineffective test [47]. Only for those markers with an AUC^ROC^≥0.75, ROC curves were used to identify new cut-off values with maximal sensitivity to detect ID maintaining the specificity as high as possible over 50%. All comparisons were made for a two-tailed significance level of 0.05. The analysis was performed using the statistical software STATA (version 12.0, STATA Corporation, College Station, TX, USA).

## Results

### Characteristics of the Study Participants

A total of 443 anaemic children were recruited as cases for the case-control study and from them, 292 (66%) underwent a bone marrow aspiration. Reasons for not performing the bone marrow aspiration were: age below 3 months in 32 (7%) cases, potential risks of sedation in 65 (15%) cases [history of seizures in 47 (11%), respiratory distress in 9 (2%) and other potential risks in 9 (2%)], adverse effects of sedation in 1 (0.2%) case, technical problems that did not allow the bone marrow aspiration in 47 (11%) cases, and parental withdraw of consent in 6 (1%) cases. Of those children with a bone marrow sample, bone marrow iron content could not be assessed in 112 (38%) cases because of absence of marrow fragments in the bone marrow smears. Thus, the analysis is restricted to the 180 (62%) cases with bone marrow smears assessable for iron content.

Children included in the analysis had an average age, gender distribution and mean Hb concentration similar to that of children who were not included. The mean ages of children included and not included in the study were (mean±SD) 22.06±13.67 and 19.97±13.56 months, respectively (p = 0.1229); the percentage of males was 57% and 59%, respectively (p = 0.6421); and the mean Hb concentrations were (mean±SD) 7.73±1.97 g/dl and 7.85±2.03 g/dl, respectively (p = 0.5584).

Demographic and clinical characteristics of the study participants are shown in table 1. Eighty percent (144/180) of the children had iron stores deficiency on the basis of bone marrow iron content. Sixty six per cent (119/180) had moderate anaemia, 25% (45/180) had severe anaemia and 9% (16/180) had very severe anaemia. An inflammatory process defined by a CRP≥1 mg/dl was detected in 88% (155/176) of the study participants. Forty four per cent (74/170) of children had *P. falciparum* infection and 43% (73/170) had clinical malaria. An EBV infection was detected in 31% (56/180) of the children, PV-B19 was found in 8% (15/180), 24% (40/164) were HIV positive and 8% (13/173) had bacteraemia. Seventy eight per cent (32/41) of the children were α-thalassaemia carriers.

### Iron Deficiency Assessment with Iron Markers

Prevalence of iron stores deficiency by the different markers according to their internationally accepted normal levels is shown in table 2. The proportion of children classified as iron deficient ranged from 1% using plasma transferrin or ferritin by age, to 77% using transferrin saturation. When plasma ferritin was used, the prevalence of ID was 1%, 9% and 12%, depending on whether age, CRP levels or none of them were considered, respectively. TIBC was associated with the lowest ID prevalence after plasma ferritin and transferrin (Table 2).

Table 3 shows the sensitivity and specificity of the different iron markers using the internationally accepted cut-off values and iron content in the bone marrow as reference. The iron markers with the lowest sensitivities were plasma ferritin (15%, 11% when combined with CRP, and 1% when combined with age), transferrin (1%) and TIBC (17%). TfR-F index and MCHC had lower sensitivities (42% and 51%, respectively) than specificities (91% and 71%, respectively), while sTfR, TfR-F index by CRP, plasma iron and transferrin saturation had higher sensitivities (83%, 75%, 70% and 81%) than specificities (50%, 56%, 54% and 40%, respectively), and all four parameters showed the highest accuracies (76%, 71%, 66% and 73%, respectively).

The AUC^ROC^ for each marker are shown in Table 4. Ferritin, transferrin, sTfR, TfR-F index, transferrin saturation and TIBC had significantly higher AUC^ROC^ than 0.5. Among them, sTfR and TfR-F index showed AUC^ROC^≥0.75 (0.75 and 0.76 respectively) (Fig. 1), thus ROC curves from these two markers were used to explore new cut-off values with maximal sensitivity to identify ID. For sTfR the ROC curve showed no better cut-off than the current one of 1.76, which already had a sensitivity of 83% and a specificity of 50%. The ROC curve for TfR-F index showed that a cut-off of 0.86 instead of the current one of 1.5 (43% change) increased the sensitivity from 42% to 78% and the accuracy from 52% to 75%, but the specificity was reduced from 91% to 65%.

## Discussion

This is the first study on the evaluation of iron markers to identify ID in a high infection pressure setting among anaemic children with any degree of anaemia. The study compares iron markers to bone marrow iron content as the “gold standard”, and shows that detection of ID still remains unresolved in settings with high infection pressure, where ID is most prevalent and its diagnosis and management most needed.

In agreement with a previous report, in this study ferritin, transferrin and TIBC had the lowest sensitivities to diagnose ID [22]. The low sensitivity of ferritin is explained for being an acute phase reactant [19], and thus, its plasma concentration may not reflect the actual iron status in the presence of inflammation, which was very prevalent in the study population (88%) [19], [48]. To solve this limitation, it is usually recommended to measure another acute phase protein [such as CRP or α-1-acid glycoprotein], and to adjust the ferritin level by the presence of inflammation [49]. However, in this study the sensitivity of ferritin did not improve after adjustment by the level of CRP, which could be explained by the stabilization of ferritin levels once iron stores are exhausted [48]. The observed low sensitivities of both transferrin and TIBC may also be due to their alteration during an inflammatory process [19], [50]. Transferrin is an acute negative protein, i.e., it decreases during an inflammatory process, while TIBC values derive from the measurement of transferrin and therefore are also affected by inflammation.

The TfR-F index has been suggested as a useful parameter for the identification of iron depletion even in settings with high infection pressure [18], and it was shown to be the best predictor of bone marrow iron stores deficiency in a previous report [22]. In contrast, in this study the TfR-F index showed a low sensitivity (42%), and only its adjustment by the level of CRP [44] increased the sensitivity to 75%, while reducing the specificity from 91% to 56%.

We found that sTfR, TfR-F index (adjusted by the level of CRP), and transferrin saturation showed the highest sensitivities. Moreover, sTfR and TfR-F index showed the highest AUC^ROC^ (≥0.75). The sTfR ROC curve indicated that there was no alternative cut-off with higher sensitivity than that of the current one (1.76 mg/l) without lowering the specificity below 50%. For the TfR-F index, the ROC curve showed that the sensitivity of this marker could be improved from 42% to 78% by changing the current cut-off from 1.5 to 0.86. It can be noticed that the performance of TfR-F index with the cut-off of 0.86 is similar to the performance of TfR-F index corrected by the CRP level (1.5 if CRP<1 mg/dl; 0.8 if CRP≥1 mg/dl). However, this similarity is not coincidental, since 88% of the study participants had a CRP≥1 mg/dl. This observation is in contrast with that of a previous study, whereby in spite of a similar prevalence of inflammation (89%) it was found that the TfR-F index unadjusted by the CRP level was a good marker of ID [22]. The findings of the current study show that the TfR-F index should be adjusted by the CRP level for maximal prediction of bone marrow iron stores deficiency in our setting, and indicate a lack of consistency of the diagnostic efficiency of current iron markers across different populations.

In this study, the MCHC, which could be a potentially feasible iron marker for resource poor settings, had an AUC^ROC^ of only 0.59 (p = 0.3382). This finding is also in contrast with the performance of this marker observed in the Malawian study where the AUC^ROC^ of MCHC was 0.68 (p = 0.001) [22]. The poor performance of MCHC in our study could be due to the high prevalence of α-thalassaemia in this population (64% among the 121 anaemic children in the case-control study; 78% among the 41 study participants included in this analysis). It has been reported that α-thalassaemia carriers have lower MCHC than non-carriers, making this marker not suitable to detect hypoferraemia in this group [51].

Differences in the participant’s selection criteria between the Malawian study and the present one may explain the discrepancies observed in the performance of the different iron markers studied. In the aforementioned study only severely anaemic children were included (Hb<5 g/dl), which may preclude its general applicability to the majority of anaemic children who do not have severe anaemia. In the present study all children with anaemia of any degree were recruited (Hb<11 g/dl). They were children with clinical conditions that required hospital admission and for whom investigation of anaemia is recommended in other less resource-limited settings. The physiopathology of anaemia may vary by its severity [52], and this may be reflected in different inflammatory processes and rates of erythropoiesis, which may have distinct effects on the iron markers evaluated.

The findings of this study show that the majority (80%) of the anaemic children were iron deficient by direct assessment of iron stores, and that sTfR and TfR-F index adjusted by CRP are the most sensitive markers with specificities of at least 50% to identify ID in this study population. However, even with these markers, 17% and 25% of children, respectively, will not be diagnosed of ID and therefore adequately treated. The fact that the children included in the study were those attending the hospital may limit the extrapolation of the findings to children in the community. However, obvious ethical reasons would not have allowed to perform bone marrow aspirations in healthy (though may be iron-deficient) children; on the other hand, children attending the hospital with anaemia are likely to be those with the greatest need to be diagnosed and adequately treated.

In summary, even the best indirect indicators of ID not only failed to detect an important proportion of iron-deficient cases, but also their assessment is not feasible in most developing settings where the majority of ID occurs. Thus, more reliable, affordable, and easy to measure iron markers are urgently needed to reduce the burden of ID anaemia in resource-poor settings where it is more frequent and severe.
